# Efficacy and safety of immune checkpoint inhibitors for brain metastases of non-small cell lung cancer: a systematic review and network meta-analysis

**DOI:** 10.3389/fonc.2025.1513774

**Published:** 2025-04-16

**Authors:** Bin Liu, Jie Chen, Mingqi Luo

**Affiliations:** ^1^ Department of Pharmacy, Hubei Cancer Hospital, Tongji Medical College, Huazhong University of Science and Technology, Wuhan, China; ^2^ School of Biological Engineering, Wuhan Polytechnic, Wuhan, China; ^3^ Department of Infectious Diseases, Zhongnan Hospital of Wuhan University, Wuhan, China

**Keywords:** immune checkpoint inhibitors, non-small cell lung cancer, brain metastases, systematic review, network meta-analysis

## Abstract

**Background:**

Previous studies have demonstrated that immune checkpoint inhibitors (ICIs) significantly improve prognosis in lung cancer patients with brain metastases (BMs). This systematic review and network meta-analysis aims to evaluate the efficacy and safety of 10 ICIs recommended by the 2024 Chinese Society of Clinical Oncology guidelines for treating non-small cell lung cancer (NSCLC) without driver genes, focusing on NSCLC patients presenting with BMs.

**Materials and methods:**

A comprehensive literature search of PubMed, Embase, and the Cochrane Library was conducted through June 2024 to identify eligible controlled trials and head-to-head randomized controlled trials investigating 10 ICIs in NSCLC patients with BMs. Pairwise and network meta-analyses were performed using hazard ratios (HRs) and relative risks (RRs) with 95% confidence intervals (CIs). Treatment efficacy was ranked hierarchically through the surface under the cumulative ranking curve (SUCRA).

**Results:**

Sixteen trials from 11 studies, encompassing 1,274 NSCLC patients with BMs, were included. The meta-analysis demonstrated that ICIs significantly improved overall survival (OS: HR, 0.66; 95% CI, 0.52–0.85; *P* = 0.001) and progression-free survival (PFS: HR, 0.67; 95% CI, 0.54–0.84; *P* < 0.001). SUCRA ranking identified pembrolizumab as the most effective agent for OS improvement (SUCRA 71%), while camrelizumab showed superior PFS benefits (SUCRA 92%). ICIs were associated with increased objective response rates (RR: 1.52; 95% CI, 1.13–2.06; *P* = 0.006), but elevated risks of immune-mediated adverse events (RR: 2.50; 95% CI, 1.46–4.30; *P* = 0.001) and grade 3–5 immune-mediated adverse events and infusion reaction (RR: 6.39; 95% CI, 1.53–26.69; *P* = 0.011).

**Conclusion:**

ICIs demonstrate superior survival benefits compared to chemotherapy in NSCLC patients with BMs, with pembrolizumab and camrelizumab emerging as optimal choices for OS and PFS improvement, respectively. However, vigilant monitoring of immune-mediated adverse events and infusion reactions remains critical in clinical practice.

## 1 Introduction

Non-small cell lung cancer (NSCLC) represents the predominant histological subtype of lung cancer, comprising 80%–85% of all cases (1). At initial diagnosis, most NSCLC patients exhibit established distant metastases in organs such as the brain, liver, and bones. Consequently, surgical intervention is generally contraindicated in advanced stages. Notably, up to 40% of stage IV NSCLC patients present with brain metastases (BMs) at diagnosis (2), a condition associated with a dismal prognosis, severely compromised quality of life, and median survival of merely 1–3 months without immediate treatment (3–5). Recent advances in multimodal therapies—including surgery, radiotherapy, pharmacotherapy, and particularly targeted therapies—have improved the clinical outcomes for NSCLC patients with BMs, extending survival and enhancing quality of life. Nevertheless, challenges persist due to treatment resistance and post-radiotherapy recurrence or metastasis (3), underscoring the urgent need for more effective therapeutic strategies.

Current first-line treatments for advanced NSCLC lacking actionable genetic alterations involve immune checkpoint inhibitors (ICIs) as monotherapy or combination regimens. These agents target key immune regulators, including programmed cell death protein 1 (PD-1), its ligand PD-L1, and cytotoxic T-lymphocyte-associated protein 4 (CTLA-4) (6–8). However, the efficacy of ICIs in BM management remains poorly understood, as systemic therapies face limited blood–brain barrier penetration. This anatomical constraint reinforces the concept of the central nervous system as an immune-privileged sanctuary with attenuated immune responses (9, 10). While prior meta-analyses have demonstrated ICI-induced improvements in overall survival (OS) and progression-free survival (PFS) for NSCLC patients with BMs (11, 12), the optimal regimen selection remains unresolved. The 2024 Chinese Society of Clinical Oncology guidelines recommend 10 ICIs for non-driver mutation NSCLC: pembrolizumab, atezolizumab, camrelizumab, sintilimab, tiragolumab, sugemalimab, toripalimab, surituzumab, pexa-vec, and nivolumab (13). To address this clinical uncertainty, we conducted a systematic review and network meta-analysis to update existing evidence and compare the efficacy of these 10 ICIs in NSCLC patients with BMs, aiming to inform evidence-based therapeutic decision-making.

## 2 Materials and methods

### 2.1 Search strategy and selection criteria

This systematic review and network meta-analysis complied with the Preferred Reporting Items for Systematic Reviews and Meta-Analyses guidelines (14). We included randomized controlled trials (RCTs) evaluating 10 ICIs in NSCLC patients with BMs, encompassing placebo-controlled and head-to-head comparative studies. No restrictions were applied regarding publication language or status.

A comprehensive search of PubMed, Embase, and the Cochrane Library was conducted through June 2024 using the following terms: pembrolizumab, atezolizumab, camrelizumab, sintilimab, tiragolumab, sugemalimab, toripalimab, surlituzumab, pexa-vec, nivolumab, non-small cell lung cancer, brain metastases, and randomized controlled trials. Additional unpublished trials were identified via ClinicalTrials.gov (U.S. National Institutes of Health), and further potentially eligible trials were identified by manually examining the reference lists of pertinent reviews.

Two investigators independently performed literature screening and study selection, with discrepancies resolved through consensus or third-party adjudication. The inclusion criteria were as follows: 1) patients: adults (≥18 years) with histopathologically/cytopathologically confirmed NSCLC and radiologically verified BMs (via brain magnetic resonance imaging, the diagnostic gold standard); 2) interventions: monotherapy or combination regimens involving the 10 ICIs, standard chemotherapy, targeted therapy, or their combinations; 3) outcomes: the primary endpoints included OS and PFS, while the secondary endpoints contained objective response rate (ORR), treatment-related adverse events (AEs; any grade, grades 3–5), neurologic AEs, and immune-mediated AEs/infusion reactions (any grade, grades 3–5). Adverse reactions were evaluated using the National Cancer Institute Common Terminology Criteria for Adverse Events version 4.0; and 4) study design: RCTs exclusively enrolling NSCLC patients with BMs receiving ICIs.

### 2.2 Data collection and quality assessment

Extracted data included the study group’s name and year of publication, trial registration number, sample size, mean age, proportion of male patients, treatment line, proportion of Eastern Cooperative Oncology Group (ECOG) 0–1, eligible criteria for BMs, intervention, control, and reported outcomes. Methodological quality was assessed using the Cochrane Risk of Bias Tool (15), evaluating seven domains: randomization sequence, allocation concealment, blinding (participants/personnel and outcome assessors), incomplete outcome data, selective reporting, and other biases. Two authors independently performed data extraction and quality assessment, with disagreements resolved by a third reviewer.

### 2.3 Statistical analysis

Survival outcomes (OS, PFS) were analyzed using hazard ratios (HRs) with 95% confidence intervals (CIs), while dichotomous outcomes (ORR, AEs) were assessed via relative risks (RRs) with 95% CIs. The summary results were analyzed utilizing a random-effects model, which accounted for the anticipated heterogeneity among the included studies (16, 17). Heterogeneity was quantified using the *I* ^2^ statistic and Cochran’s Q test, with *I* ^2^ >50% or Q test *P <*0.10 indicating significant heterogeneity (18). Sensitivity analyses tested result stability by sequentially excluding individual studies (19). Subgroup analyses stratified by treatment line, BM eligibility criteria, and intervention type were conducted for OS/PFS, with interaction *P*-values assessing subgroup differences (20).

A Bayesian network meta-analysis integrated direct and indirect comparisons to rank ICI efficacy (21). Loop inconsistency was evaluated via node-splitting methods (22), while global consistency was verified using the design-by-treatment interaction model (21). Treatment hierarchies were established using surface under the cumulative ranking curve (SUCRA) values (23). Additionally, pairwise comparison analyses were performed for every outcome measure under investigation. Publication bias was assessed via comparison-adjusted funnel plots (24) and quantified using Egger’s/Begg’s tests for OS/PFS (25, 26). All reported *P*-values were two-tailed, and the significance level was set at 0.05. The statistical analyses were conducted using the STATA software package (version 12.0; StataCorp LLC, College Station, TX, USA).

## 3 Results

### 3.1 Literature search and study selection

**Figure 1:**
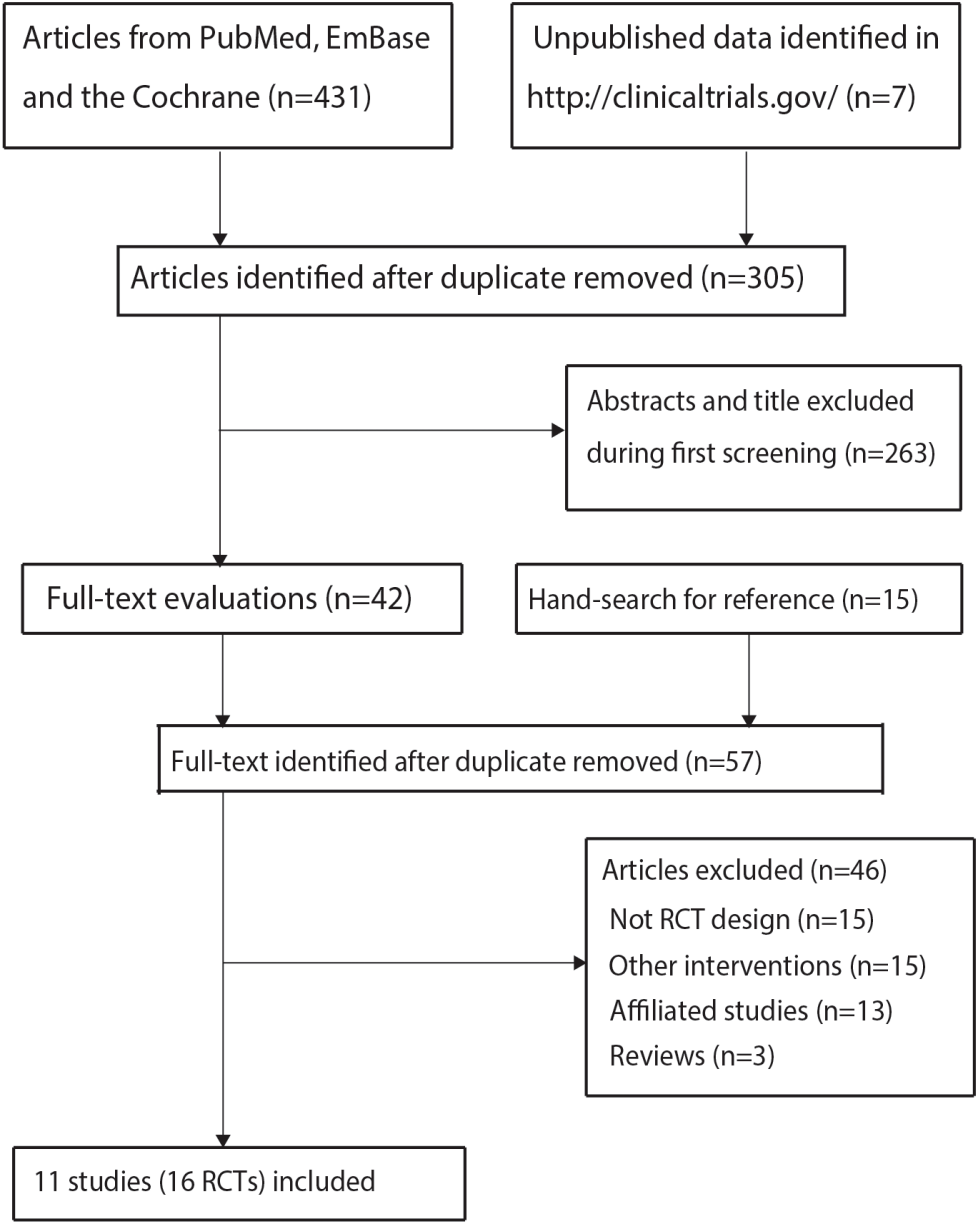
The PRISMA flowchart for literature search and study selection.

The initial database search yielded 431 records, with 126 duplicates removed. After excluding 263 irrelevant articles through title/abstract screening, 42 full-text articles were assessed. Thirty-one studies were further excluded: 15 for non-relevant interventions, 13 as substudies, and 3 reviews. Manual reference screening identified 15 additional articles, all excluded due to observational designs. Ultimately, 16 trials from 11 unique studies met the inclusion criteria (27–37). The study selection process is detailed in **Figure 1**.

### 3.2 Characteristics of the included studies

**Table 1** presents the baseline characteristics of the trials included and the profiles of the participating patients. In total, these trials encompassed 1,274 NSCLC patients who had developed BMs, with individual study populations ranging in size from 15 to 293 participants. Seven trials included patients who received first-line therapies, while the remaining four studies included patients who received second-line therapies. Five studies investigated the therapeutic effect of nivolumab, two studies reported the therapeutic effect of pembrolizumab, two studies investigated the therapeutic effect of sintilimab, and the remaining two studies reported the therapeutic effects of atezolizumab and camrelizumab, respectively. The summary of the methodological quality for each trial included is presented in **Table 2**, and the overall quality of the included studies was moderate to high.

**Table 1:** The baseline characteristics of the included trials and involved patients.

Study	Trial no.	Sample size	Mean age (years)	Male (%)	Treatment line	ECOG 0–1 (%)	Eligible criteria for BMs	Intervention	Control
CheckMate 057 2015 (27)	NCT01673867	68 (34/34)	NA	NA	2	100.0	Treated and stable	Nivolumab (3 mg/kg every 2 weeks)	Docetaxel (75 mg/m^2^ body-surface area every 3 weeks)
OKY 2019 (28)	NCT02008227	123 (61/62)	60.8	54.5	2	100.0	Asymptomatic, treated	Atezolizumab (1,200 mg every 3 weeks)	Docetaxel (75 mg/m^2^ body-surface area every 3 weeks)
CheckMate 078 2019 (29)	NCT02613507	72 (45/27)	NA	NA	2	100.0	Treated and stable	Nivolumab (3 mg/kg every 2 weeks)	Docetaxel (75 mg/m^2^ body-surface area every 3 weeks)
CheckMate 227 2019 (30)	NCT02477826	115 (64/51)	NA	NA	1	100.0	Treated and stable	Nivolumab (360 mg every 3 weeks)	Platinum-doublet chemotherapy (up to 4 cycles every 3 weeks)
ORIENT-11 2020 (31)	NCT03607539	58 (36/22)	61.0	76.6	1	100.0	Asymptomatic	Sintilimab (200 mg) plus pemetrexed and platinum (up to 4 cycles every 3 weeks)	Pemetrexed and platinum (up to 4 cycles every 3 weeks)
ONO-4538-52/TASUKI-52 2021 (32)	NCT03117049	77 (36/41)	NA	NA	1	100.0	Treated and stable	Nivolumab plus carboplatin, paclitaxel, and bevacizumab (up to 6 cycles every 3 weeks)	Carboplatin, paclitaxel, and bevacizumab (up to 6 cycles every 3 weeks)
Camel 2021 (33)	NCT03134872	15 (10/5)	NA	NA	1	100.0	Untreated	Camrelizumab (200 mg) plus carboplatin and pemetrexed (up to 4–6 cycles every 3 weeks)	Carboplatin and pemetrexed (up to 4–6 cycles every 3 weeks)
CheckMate 9LA 2021 (34)	NCT03215706	122 (64/58)	NA	NA	1	100.0	Treated and stable	Nivolumab (360 mg every 3 weeks) plus ipilimumab (1 mg/kg every 6 weeks) plus (up to 2 cycles every 3 weeks)	Platinum-doublet chemotherapy (up to 4 cycles every 3 weeks)
KEYNOTE-001, 010, 024, 042 2021 (35)	NCT01295827; NCT01905657; NCT02142738; NCT02220894	293 (199/94)	59.3	51.9	1	99.7	Treated and stable	Pembrolizumab (200 mg every 3 weeks)	Docetaxel or platinum-based chemotherapy (4–6 cycles)
KEYNOTE-021, 189, 407 2021 (36)	NCT02039674; NCT02578680; NCT02775435	171 (105/66)	63.2	62.0	1	100.0	Treated and stable	Pembrolizumab (200 mg every 3 weeks) plus platinum-based chemotherapy (4 cycles)	Platinum-based chemotherapy (4 cycles)
ORIENT-31 2022 (37)	NCT03802240	160 (105/55)	NA	NA	2	100.0	Treated and stable	Sintilimab (200 mg) plus premetrexed (500 mg/m^2^ body-surface area) and cisplatin (75 mg/m^2^ body-surface area)	Premetrexed (500 mg/m^2^ body-surface area) and cisplatin (75 mg/m^2^ body-surface area)

NA, not available.

**Table 2:** The methodological quality assessment of the included trials.

Study	Random sequence generation	Allocation concealment	Blinding of participants and personnel	Blinding of outcome assessment	Incomplete outcome data	Selective reporting	Other bias
NCT01673867	Low	High	High	Low	Low	Low	Low
NCT02008227	Low	Low	High	Unclear	Low	Low	Low
NCT02613507	Low	High	High	Unclear	Low	Low	Low
NCT02477826	Low	High	High	Low	Low	Low	Low
NCT03607539	Low	Unclear	Low	Low	Low	Low	Low
NCT03117049	Low	Unclear	Low	Low	Low	Low	Low
NCT03134872	Low	High	High	Low	Low	Low	Low
NCT03215706	Low	High	High	Low	Low	Low	Low
NCT01295827	Low	Unclear	Unclear	Unclear	Low	Low	Low
NCT01905657	Low	Unclear	Unclear	Unclear	Low	Low	Low
NCT02142738	Low	Unclear	Unclear	Unclear	Low	Low	Low
NCT02220894	Low	Unclear	Unclear	Unclear	Low	Low	Low
NCT02039674	Low	Unclear	Low	Low	Low	Low	Low
NCT02578680	Low	Unclear	Low	Low	Low	Low	Low
NCT02775435	Low	Unclear	Low	Low	Low	Low	Low
NCT03802240	Low	Unclear	Low	Low	Low	Low	Low

### 3.3 Overall survival

**Figure 2:**
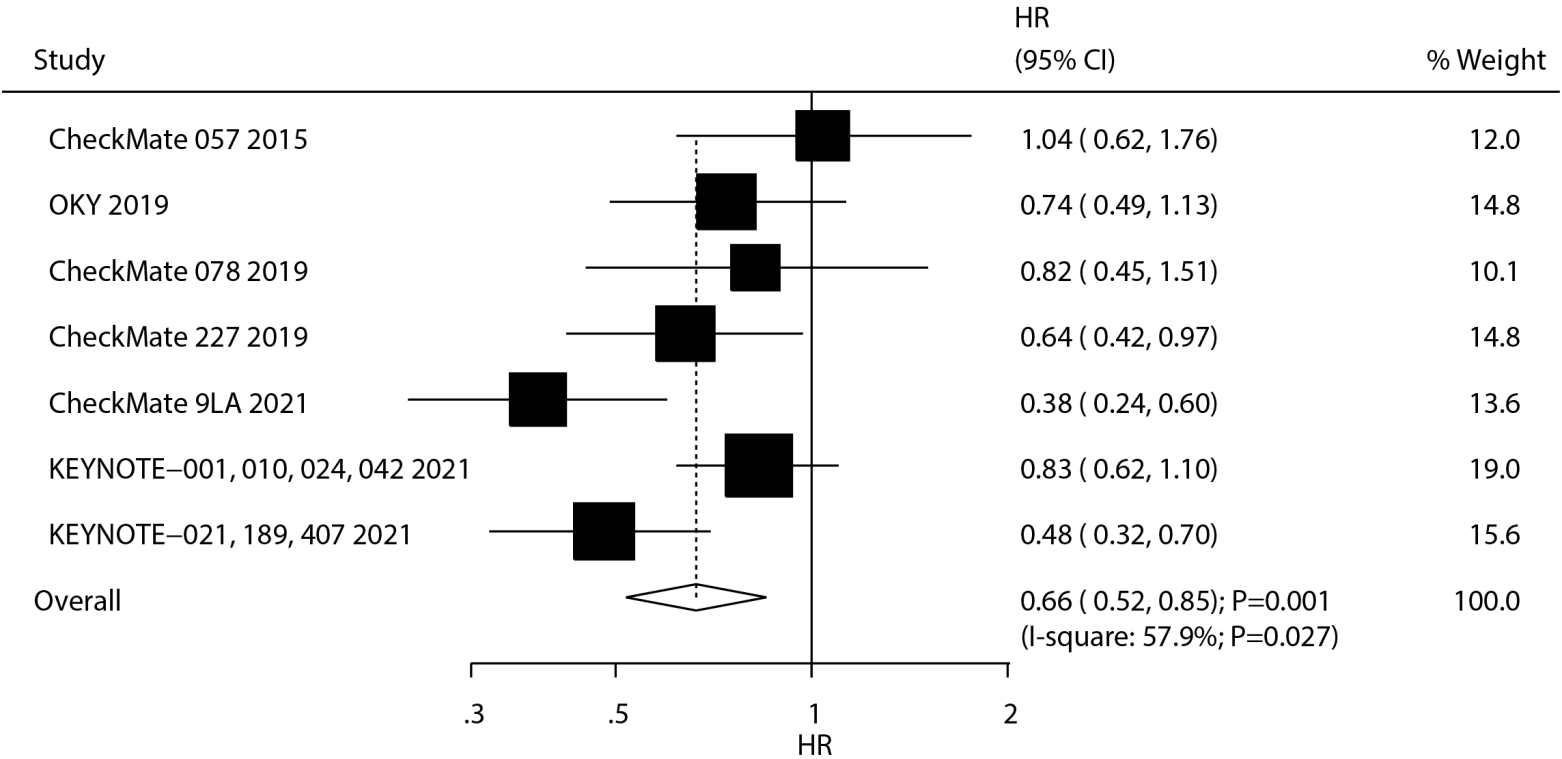
The summary result for the use of ICIs on OS in NSCLC patients with BMs.

A total of seven studies reported the therapeutic effect of ICIs on OS in NSCLC patients with BMs. We noted that ICIs were associated with an improvement in OS as compared with chemotherapy (HR: 0.66; 95% CI, 0.52–0.85; *P* = 0.001; **Figure 2**), and significant heterogeneity was observed (*I* ^2^ = 57.9%; *P* = 0.027). The sensitivity analysis revealed that the combined outcome was robust and remained unchanged even upon the exclusion of any single study, indicating a high degree of reliability in the findings (**Supplementary Figure S1**). Subgroup analyses revealed OS benefits primarily in first-line therapy recipients and patients with treated/stable BMs (**Supplementary Figures S2**-**S4**).

### 3.4 Progression-free survival

**Figure 3:**
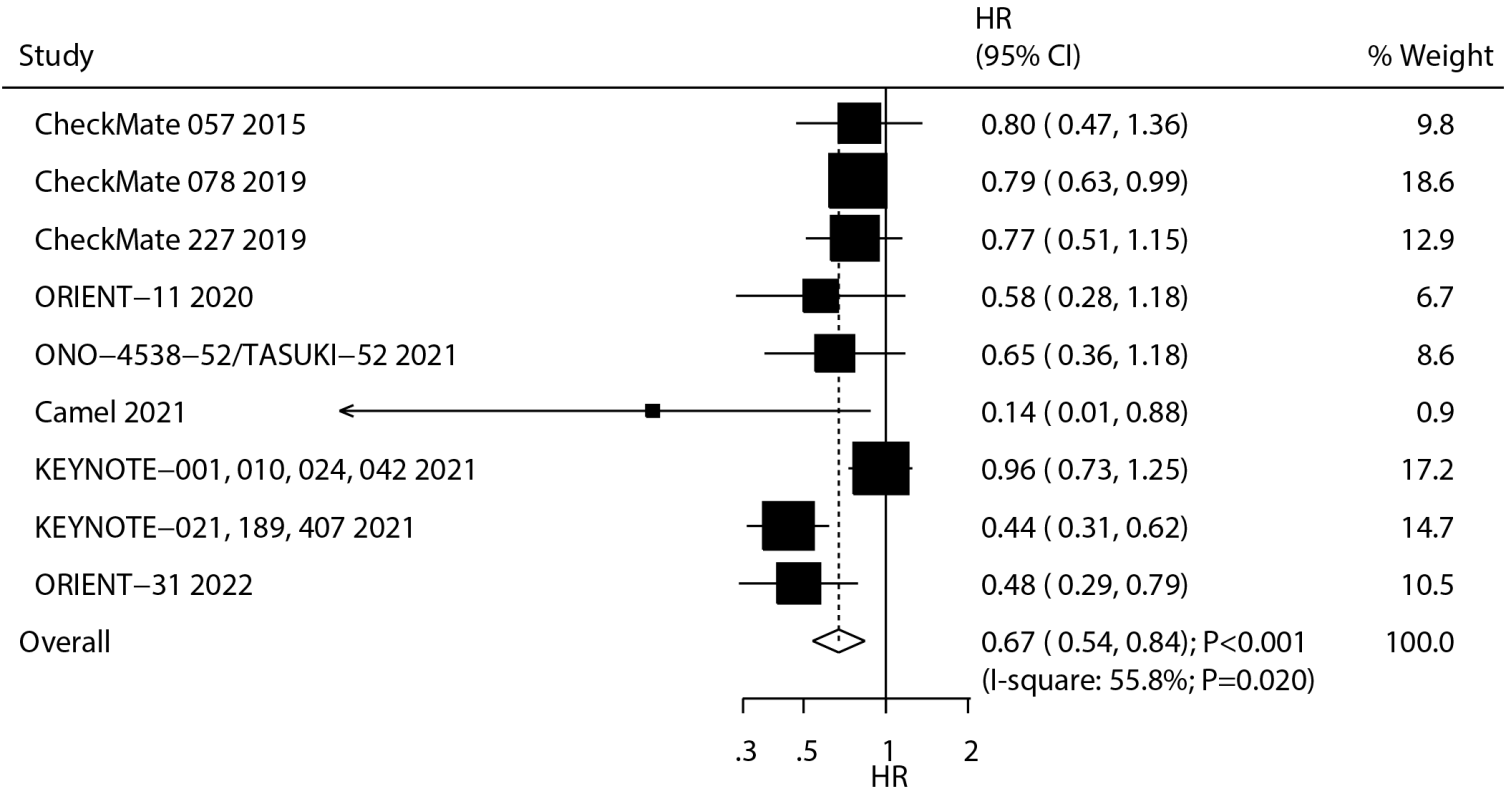
The summary result for the use of ICIs on PFS in NSCLC patients with BMs.

A total of nine studies reported the therapeutic effect of ICIs on PFS in NSCLC patients with BMs. The summary result indicated that ICIs significantly improved PFS as compared with chemotherapy (HR: 0.67; 95% CI: 0.54–0.84; *P* < 0.001; **Figure 3**), and significant heterogeneity was observed across the included studies (*I* ^2^ = 55.8%; *P* = 0.020). The pooled conclusion was stable and not affected by any single study (**Supplementary Figure S5**). Significant PFS benefits were observed with nivolumab and sintilimab and across treatment lines in patients with treated/stable BMs (**Supplementary Figures S6**-**S8**).

### 3.5 Secondary outcomes

**Figure 4:**
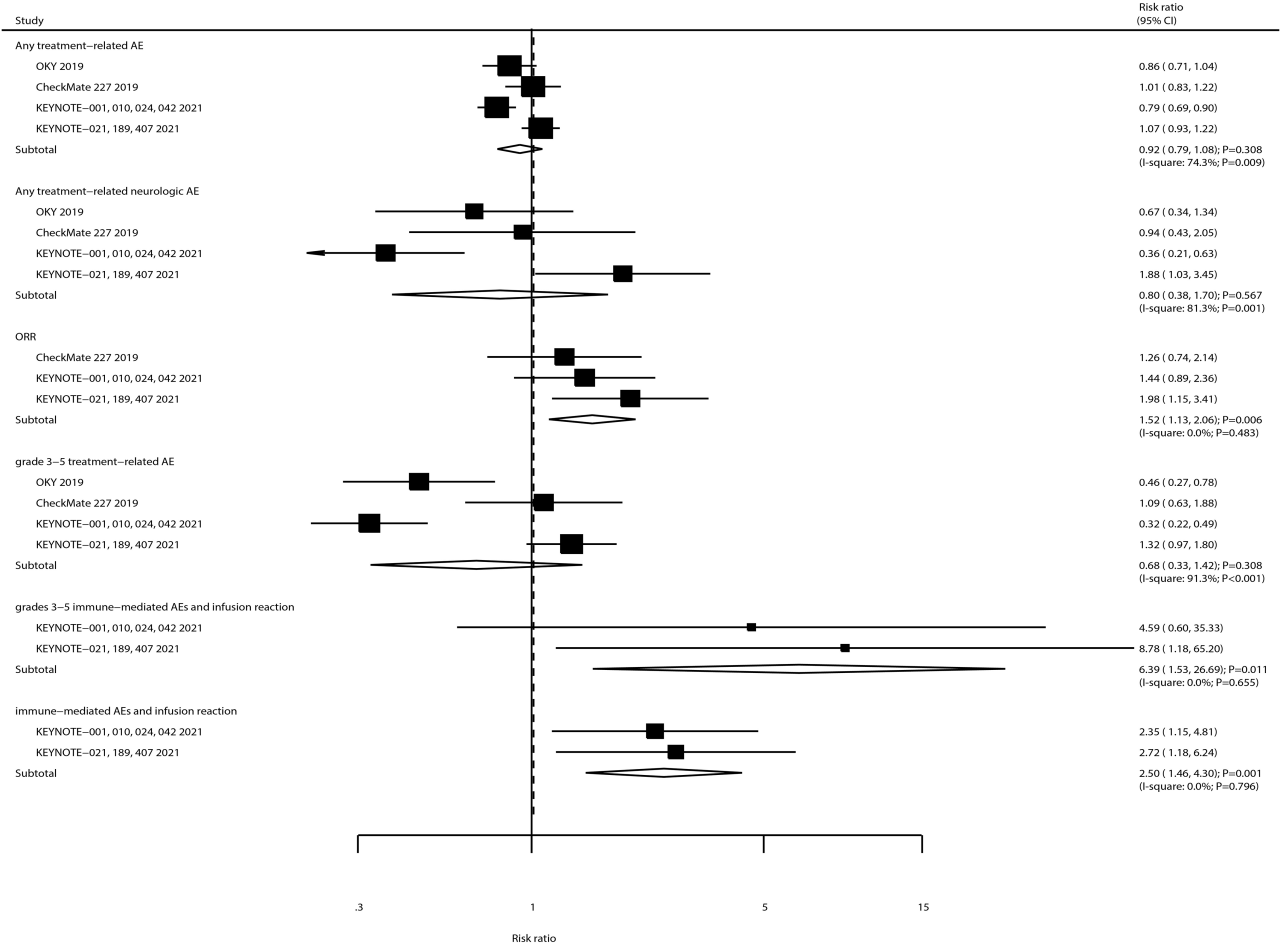
The summary result for the use of ICIs on ORR, any or grade 3–5 treatment-related AEs, any treatment-related neurologic AE, any or grade 3–5 immune-mediated AEs, and infusion reaction in NSCLC patients with BMs.

The summary results for the effects of ICIs on secondary outcomes are shown in **Figure 4**. We noted that ICIs significantly increased the incidence of ORR (RR: 1.52; 95% CI, 1.13–2.06; *P* = 0.006). Moreover, the risks of immune-mediated AEs and infusion reaction (RR: 2.50; 95% CI, 1.46–4.30; *P* = 0.001) and grade 3–5 immune-mediated AEs and infusion reaction (RR: 6.39; 95% CI, 1.53–26.69; *P* = 0.011) were significantly increased in patients receiving ICIs. Furthermore, ICIs had no significant effects on the risk of any treatment-related AE, grade 3–5 treatment-related AEs, and any treatment-related neurologic AE. There was a significant heterogeneity for any treatment-related AE (*I* ^2^ = 74.38%; *P* = 0.009), grade 3–5 treatment-related AEs (*I* ^2^ = 91.3%; *P* < 0.001), and any treatment-related neurologic AE (*I* ^2^ = 81.3%; *P* = 0.001). Finally, there was no evidence of heterogeneity for ORR (*I* ^2^ = 0.0%; *P* = 0.483), immune-mediated AEs and infusion reaction (*I* ^2^ = 0.0%; *P* = 0.796), and grade 3–5 immune-mediated AEs (*I* ^2^ = 0.0%; *P* = 0.655).

### 3.6 Network meta-analysis

**Supplementary Figure S9** illustrates the network of eligible comparisons made for OS. The size of each node is proportional to the number of trials contributing to that particular comparison, while the thickness of the connecting lines, or edges, between nodes reflects the precision of the direct estimates for every pairwise comparison. To evaluate and rank the therapeutic efficacy of ICIs on OS, SUCRA probabilities were employed. Notably, pembrolizumab emerged with relatively higher efficacy, boasting a SUCRA score of 71.0% (**Supplementary Figure S10**). Additionally, the outcomes from pairwise comparisons focusing on the occurrence of complete remission are illustrated in **Supplementary Figure S11**, and no significant difference was observed in the comparison of any two ICIs.

**Supplementary Figure S12** displays the network of valid comparisons concerning PFS. Based on SUCRA probabilities, camrelizumab was identified to provide the most favorable therapeutic impact on PFS (SUCRA: 92%; **Supplementary Figure S13**). The pairwise comparison outcomes related to PFS are detailed in **Supplementary Figure S14**, highlighting that sintilimab outperforms chemotherapy in enhancing PFS.

### 3.7 Publication bias

**Figure 5:**
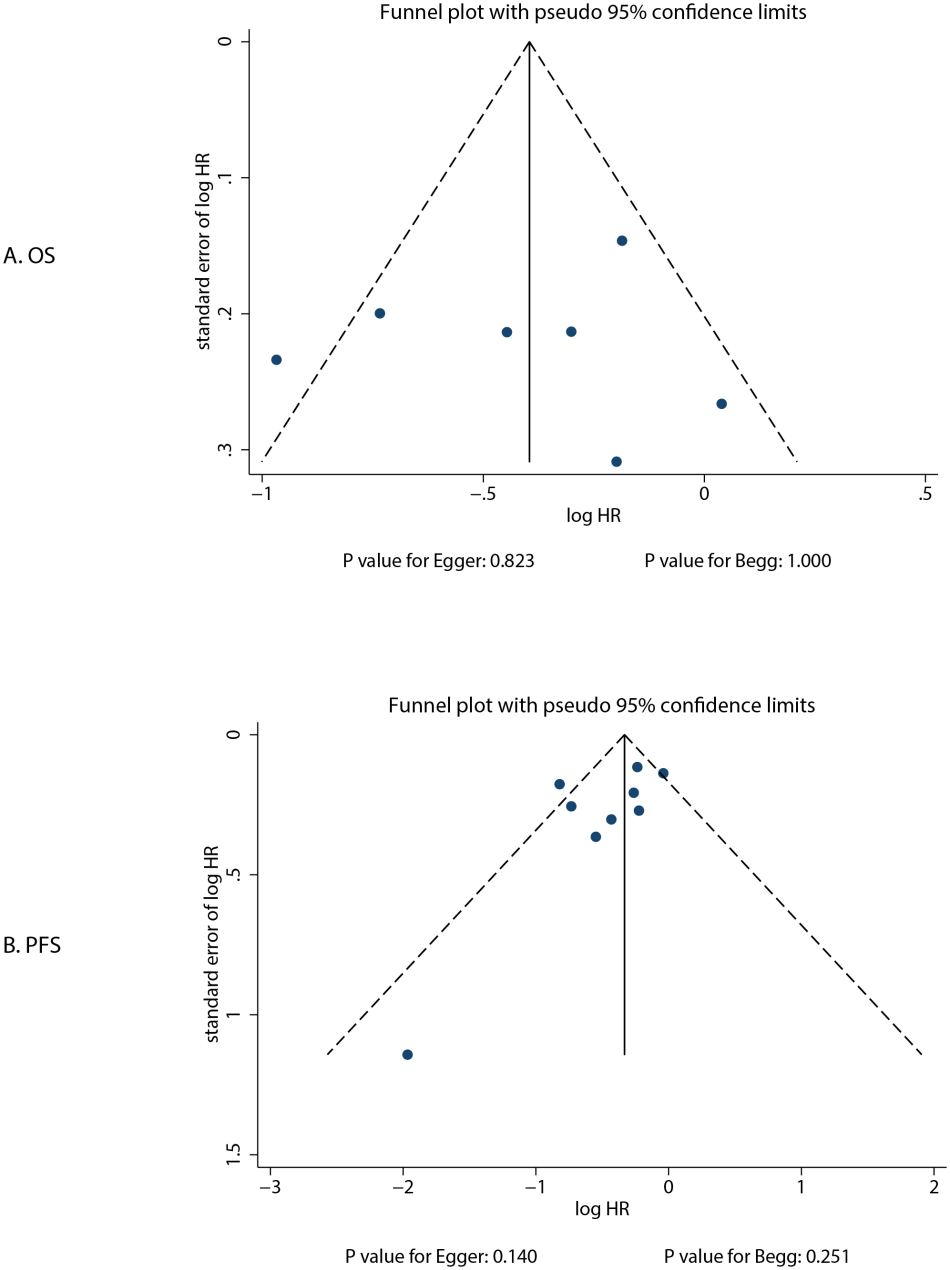
Funnel plots for OS and PFS. **(A)**: OS; **(B)**: PFS.

The review of the funnel plot could not rule out potential publication bias for OS and PFS (**Figure 5**). The Egger’s and Begg’s tests indicated no significant publication biases for OS (*P*-value for Egger: 0.823; *P*-value for Begg: 1.000) and PFS (*P*-value for Egger: 0.140; *P*-value for Begg: 0.251).

## 4 Discussion

Our systematic review and network meta-analysis offers enhanced comprehensiveness by incorporating 10 distinct ICIs and examining key clinical endpoints such as OS, PFS, ORR, and AEs. This exhaustive, quantitative assessment encompasses 16 RCTs from 11 distinct studies, involving a total of 1,274 NSCLC patients presenting with BMs. These patients were systematically allocated to receive one of 10 different ICI treatment protocols. This study found that OS and PFS were significantly improved in patients treated with ICIs, and the optimal treatments for OS and PFS were pembrolizumab and camrelizumab, respectively. Moreover, the use of ICIs was associated with increased incidences of ORR, immune-mediated AEs and infusion reaction, and grade 3–5 immune-mediated AEs and infusion reaction.

Reviewing prior systematic reviews and meta-analyses, Chen et al. identified 36 studies and found that combination therapy centered around ICIs confers a substantial long-term survival advantage to patients who are not candidates for targeted therapies. The most pronounced enhancements have been noted in increasing the intracranial ORR, as well as significantly prolonging both OS and intracranial PFS (11). However, the findings of this study, which are based on observational studies and RCTs, indicate that the level of evidence may be subject to certain limitations. Yang et al. identified 11 RCTs and found that PD-1/PD-L1 inhibitors have demonstrated significant improvement in the therapeutic effectiveness for patients suffering from BMs originating from lung cancer (12). Notably, the BM patients included in this study originated from both SCLC and NSCLC, and there may be significant prognostic differences between patients with BMs from these different origins, which could influence the efficacy of ICI treatments. Zhang et al. performed a network meta-analysis of 25 RCTs comparing the treatments for BMs from EGFR/ALK-negative/unselected NSCLC and found that ICI-based therapies, notably those incorporating ICI combinations, have demonstrated significant potential in managing previously treated BMs stemming from EGFR/ALK-negative or non-specifically selected NSCLC cases (38). However, the network constructed in this study is based on all treatment modalities, and while the results offer greater comprehensiveness, they are influenced by the varying quality of the studies and the strength of indirect comparisons, potentially introducing additional biases. Therefore, the current study was performed to compare and rank the efficacy of 10 types of ICIs for NSCLC patients with BMs.

The summary results indicated that the use of ICIs could significantly improve OS, PFS, and ORR compared with chemotherapy. Several reasons could explain these results: 1) the tumor microenvironment of BMs has immunosuppressive properties. ICIs reverse T-cell exhaustion, activate local and systemic immune responses, and break the tumor immune escape mechanism by blocking the PD-1/PD-L1 or CTLA-4 pathways (39). 2) Due to their large molecular weight, chemotherapy drugs have difficulty penetrating the blood–brain barrier, resulting in a low drug concentration in the brain. In contrast, ICIs exert their effects by activating the systemic immune system, and they can indirectly kill tumor cells without the need to directly penetrate the blood–brain barrier (40); and 3) ICIs form an immunological memory by activating T cells, which may continuously suppress tumor recurrence. In contrast, chemotherapy only kills cancer cells in the short term and lacks a long-term protective effect (41). In addition, for OS, the optimal treatment option is pembrolizumab; for PFS, the best treatment is camrelizumab. Both pembrolizumab and camrelizumab demonstrate a stronger ability to penetrate the blood–brain barrier, allowing them to reach brain tumor sites at higher concentrations and effectively target cerebral metastatic lesions (10, 42). Furthermore, pembrolizumab, in particular, incorporates the screening of biomarkers, such as PD-L1 expression levels and tumor mutation burden, to identify patient populations most likely to benefit, thereby facilitating a more precise therapeutic approach (43).

The summary results found ICIs significantly increasing the risk of any grade or grade 3–5 immune-mediated adverse events and infusion reaction. ICIs enhance antitumor immune responses by lifting the natural brakes on the immune system. Specifically, they target key immune checkpoints such as PD-1/PD-L1 and CTLA-4, thereby activating T cells and promoting their recognition and destruction of tumor cells. However, this immune activation is global in nature, not only targeting cancerous cells but also potentially misdirecting against normal tissues within the body. This leads to the immune system attacking itself, resulting in a cascade of autoimmune-like reactions, known as immune-mediated adverse events. Several mechanisms could explained these results: 1) ICIs facilitate a broad and non-specific T-cell activation, which can lead to immune responses against healthy tissues, causing inflammation and damage across multiple organ systems throughout the body, ranging from mild to severe (44); 2) unlike traditional targeted therapies, ICIs do not differentiate between normal tissue and tumor tissue. Once activated, immune cells may fail to accurately discern and may inadvertently attack normal cells, in contrast to the selective action of targeted therapies (45); 3) there are significant variations in patients’ responses to ICIs, with some individuals potentially exhibiting a more vigorous immune response, rendering them more susceptible to immune-related AEs (46); and 4) ICIs not only trigger an immediate immune response but also induce long-term immune memory (41). This can result in immune-related AEs persisting or even emerging anew after the cessation of treatment (47–49).

It is crucial to underscore the limitations of this study. Firstly, the heterogeneity of treatment regimens among the included trials may have influenced the survival outcomes for NSCLC patients with BMs. Secondly, variation in the severity of BMs resulting from NSCLC was observed across different trials. Thirdly, the 2024 CSCO guidelines recommend the use of 10 ICIs for the treatment of NSCLC. However, this study only includes five of these drugs and lacks evaluations of the efficacy and safety of the other five ICIs in treating BMs from NSCLC. Fourthly, the paucity of trials reporting on several secondary endpoints led to inconclusive and less robust findings due to the small sample size, such as only two trials reporting the risk of immune-mediated adverse events and infusion reaction. Fifthly, there are differences in the acceptability of immune-related toxicity between Asian and Caucasian populations (50). However, due to the limited number of relevant studies included in this research, we were unable to fully balance the proportion of Asian and Caucasian populations in the study. This may affect the generalizability of the research results among different ethnic groups. Sixth, the BM subgroup analyses in all included studies were exploratory, which could introduce a risk of selection bias because there may be a non-random selection of patients for the BM subgroup analysis. Lastly, the study faces inherent constraints typical of meta-analyses derived from published literature, such as potential publication bias and limitations in conducting in-depth analyses due to data accessibility issues.

## 5 Conclusion

This systematic review and network meta-analysis demonstrates the superior efficacy of ICIs over chemotherapy in improving OS and PFS for NSCLC patients with BMs. Pembrolizumab and camrelizumab emerged as optimal agents for OS and PFS enhancement, respectively. While ICIs significantly increased ORR, they also elevated the risks of immune-mediated AEs and infusion reactions across all grades and grade 3–5 toxicities. These findings underscore the necessity for vigilant monitoring of immune-related toxicities during ICI administration in this patient population.

## Data Availability

The original contributions presented in the study are included in the article/**Supplementary Material**. Further inquiries can be directed to the corresponding author.
