# Supplementary material for: Efficacy and safety of immune checkpoint inhibitors for brain metastases of non-small cell lung cancer: a systematic review and network meta-analysis
Source: Front Oncol. 2025 Apr 16;15:1513774. doi: 10.3389/fonc.2025.1513774 (PMC12040931; doi:10.3389/fonc.2025.1513774)
Supplement: Supplementary file 1 [file DataSheet1.pdf]

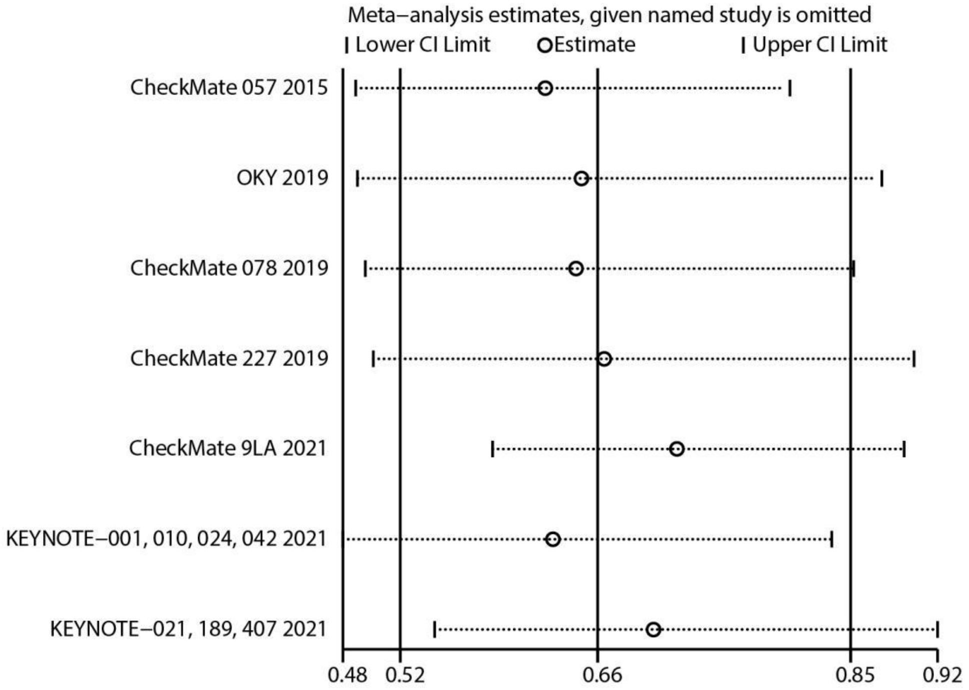

Figure S1. Sensitivity analysis for OS

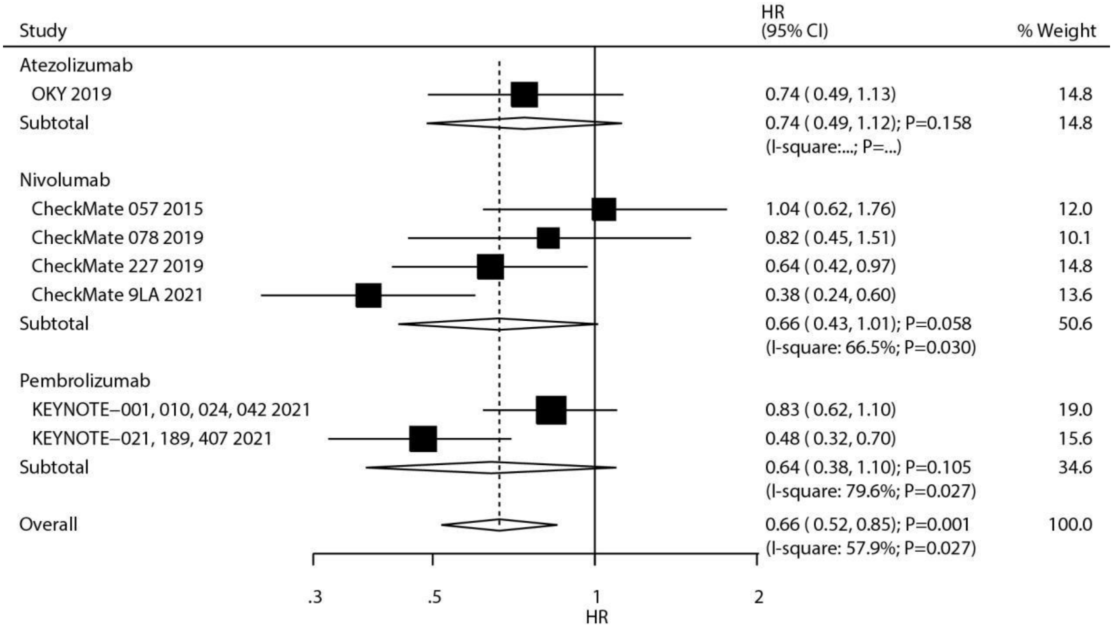

Figure S2. Subgroup analysis for OS according to intervention

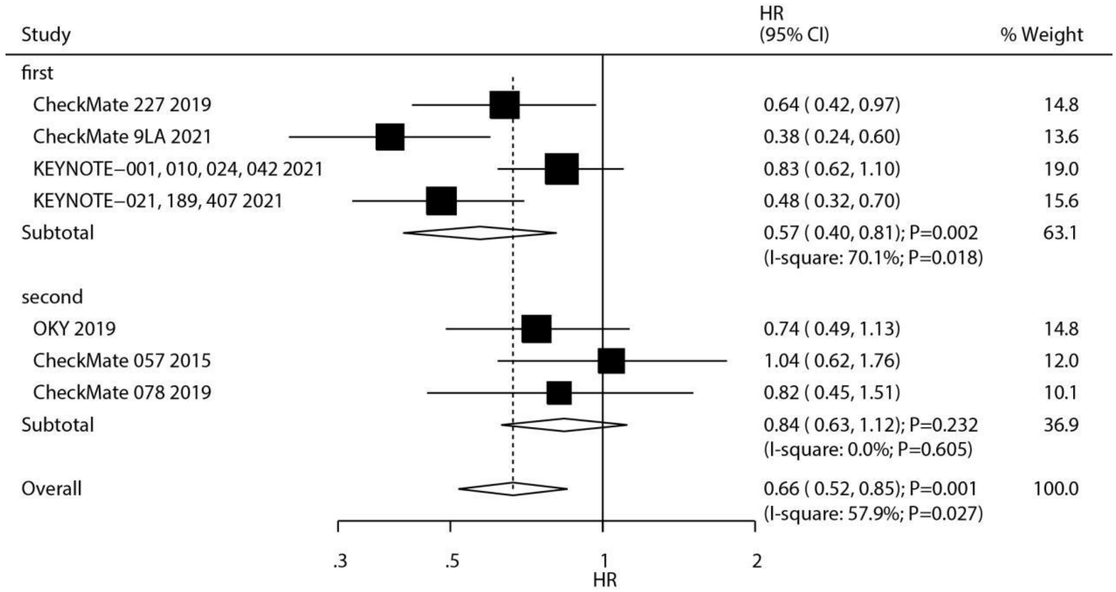

Figure S3. Subgroup analysis for OS according to treatment line

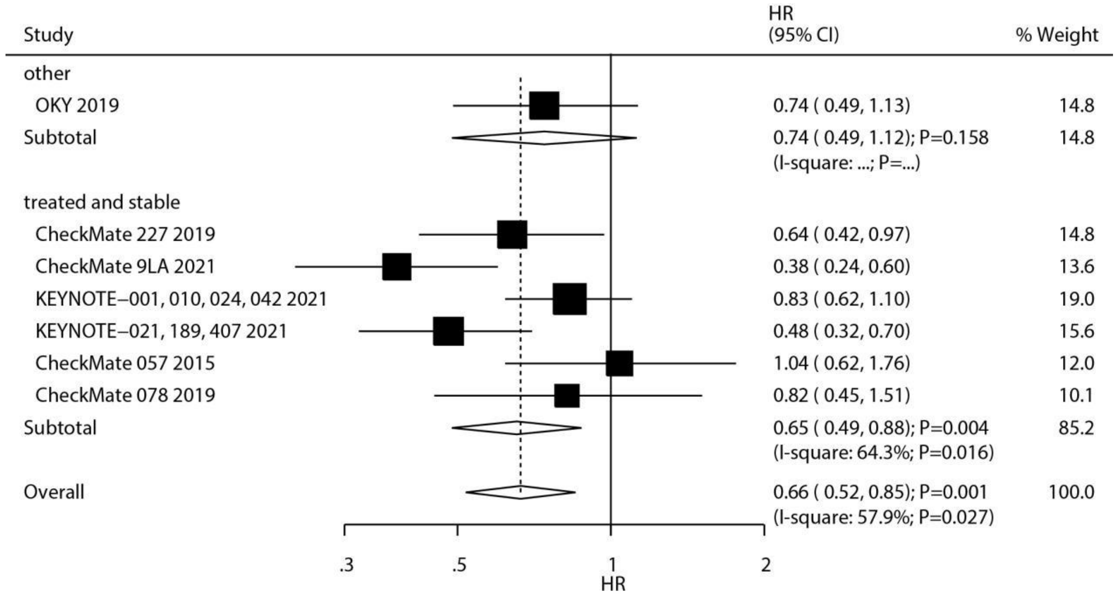

Figure S4. Subgroup analysis for OS according to eligible criteria for BMs

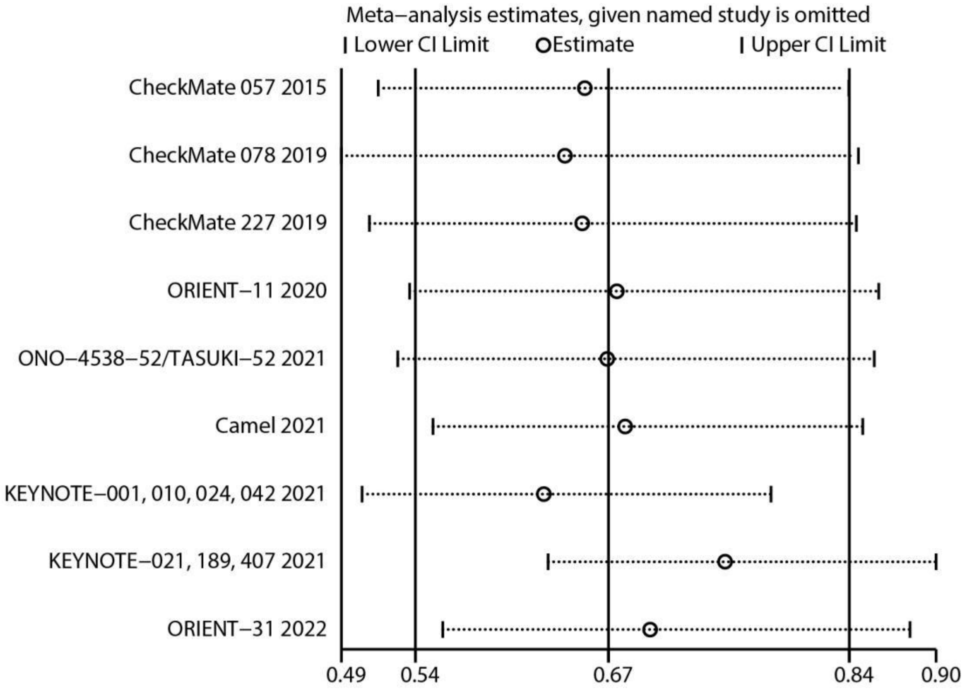

Figure S5. Sensitivity analysis for PFS

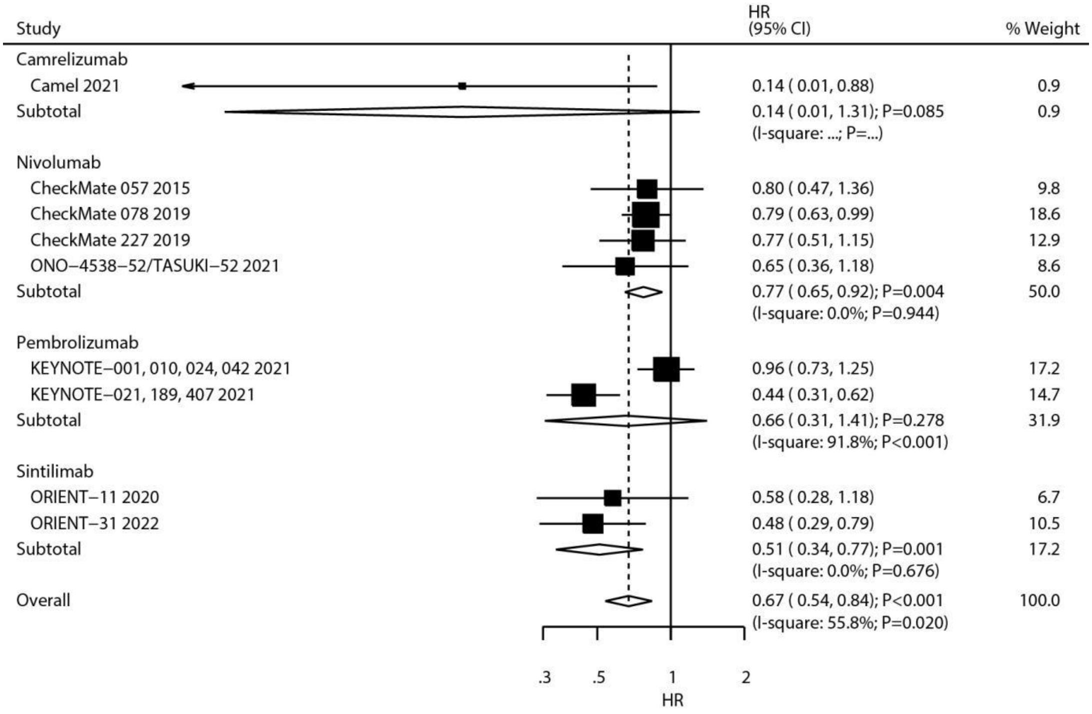

Figure S6. Subgroup analysis for PFS according to intervention

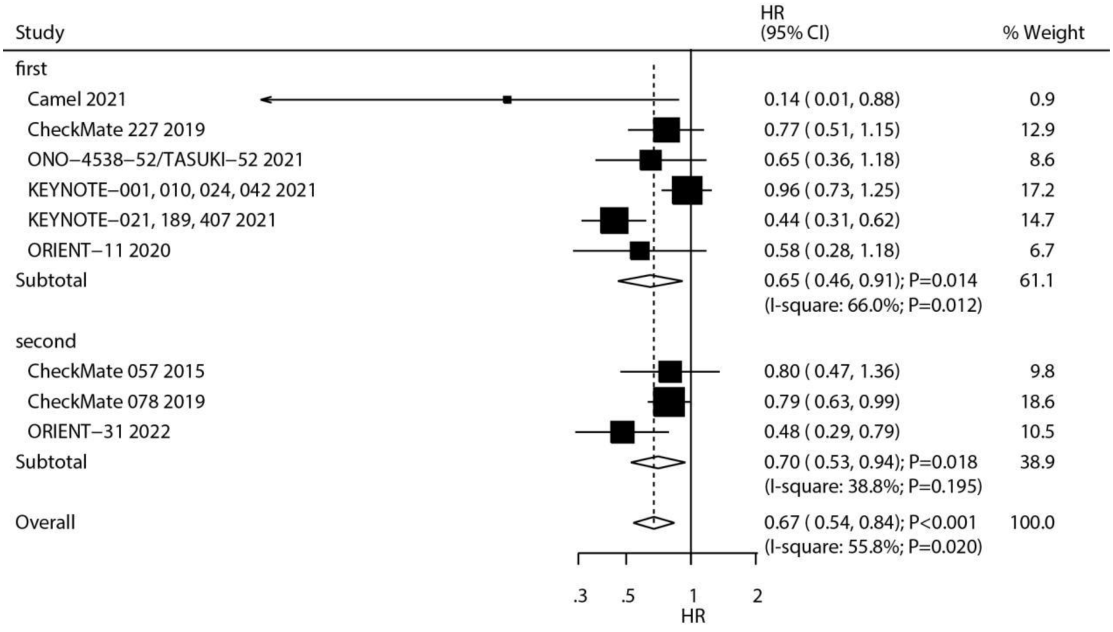

Figure S7. Subgroup analysis for PFS according to treatment line

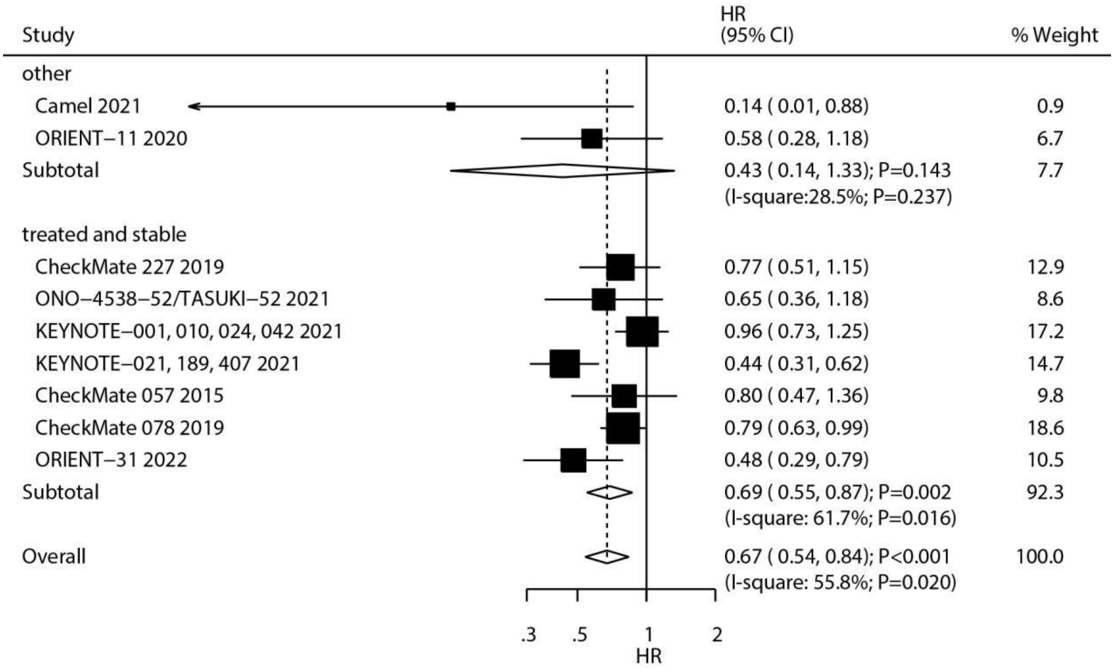

Figure S8. Subgroup analysis for PFS according to eligible criteria for BMs

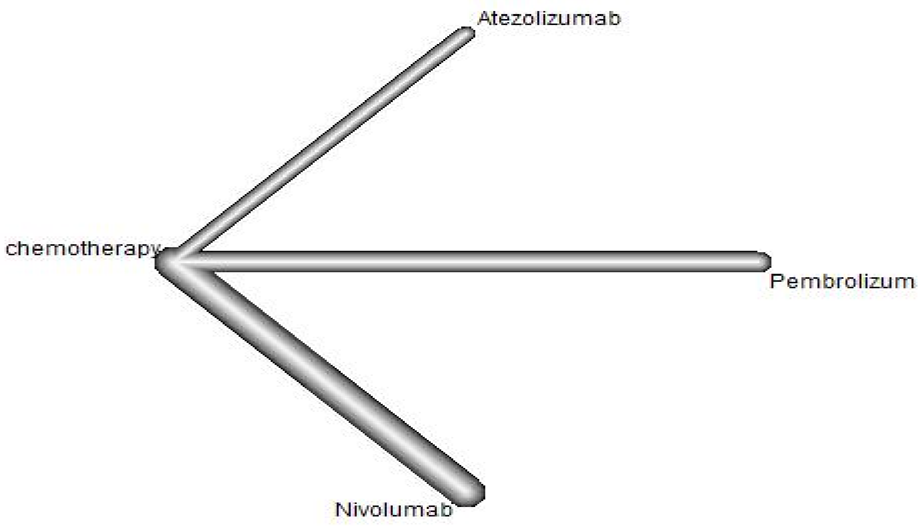

Figure S9. Network of comparisons for OS included in the analysis.

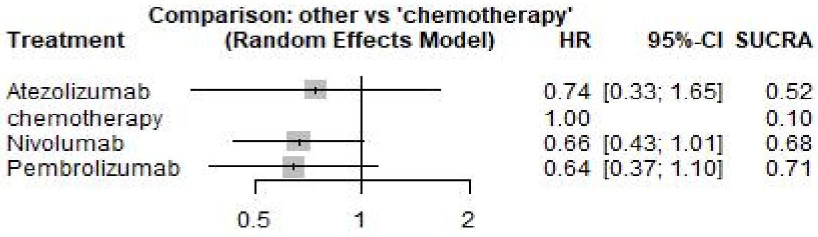

Figure S10. The SUCRA rank test for OS.

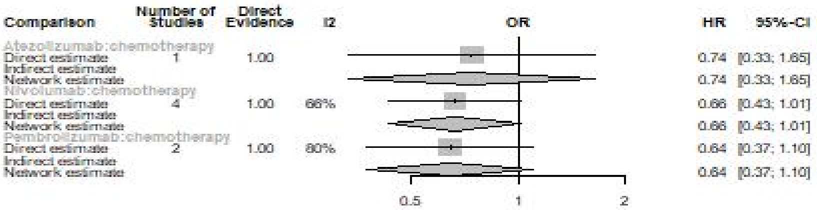

Figure S11. The pair-wise comparisons agents for OS.

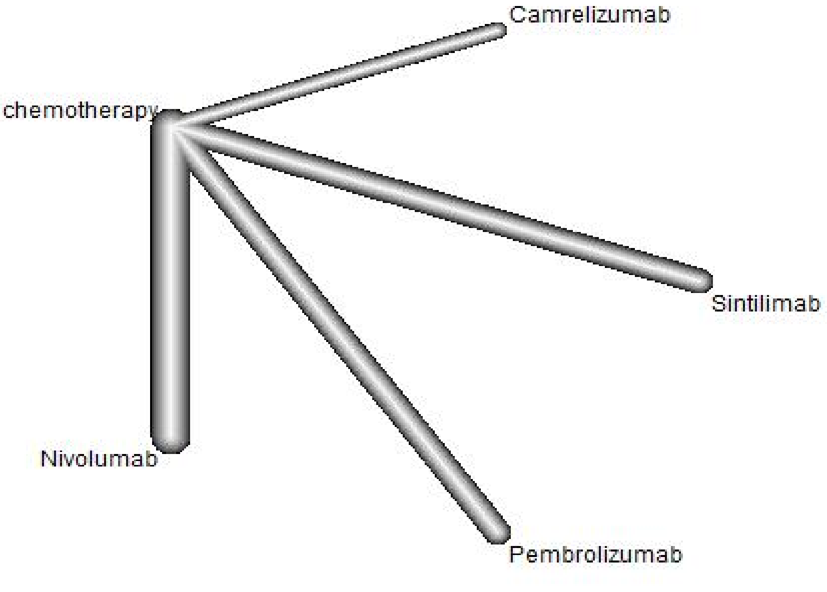

Figure S12. Network of comparisons for PFS included in the analysis.

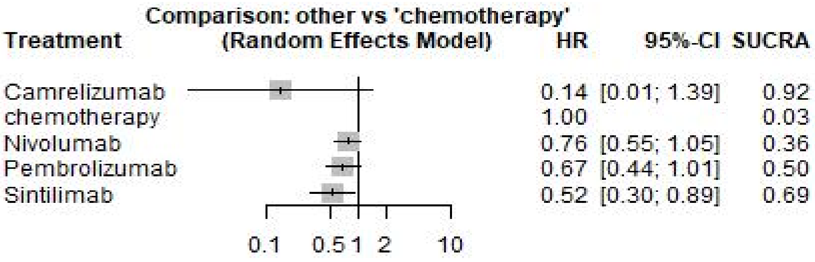

Figure S13. The SUCRA rank test for PFS.

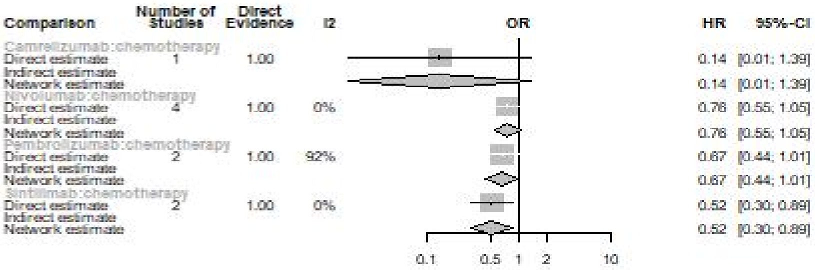

Figure S14. The pair-wise comparisons agents for PFS.
